# Administration of DHA Reduces Endoplasmic Reticulum Stress-Associated Inflammation and Alters Microglial or Macrophage Activation in Traumatic Brain Injury

**DOI:** 10.1177/1759091415618969

**Published:** 2015-12-16

**Authors:** Lloyd D. Harvey, Yan Yin, Insiya Y. Attarwala, Gulnaz Begum, Julia Deng, Hong Q. Yan, C. Edward Dixon, Dandan Sun

**Affiliations:** 1 Department of Neurology, University of Pittsburgh, Pittsburgh, PA, USA; 2 Department of Neurology, the Second Hospital of Dalian Medical University, Dalian, China; 3 Department of Neurosurgery, Brain Trauma Research Center, University of Pittsburgh, Pittsburgh, PA, USA; 4 Geriatric Research, Education and Clinical Center, Veterans Affairs Pittsburgh Health Care System, Pittsburgh, PA, USA

**Keywords:** cortical contusion injury, docosahexaenoic acid, microglial polarization, neuroinflammation, nuclear factor kappa-light-chain-enhancer of activated B cells, secondary injury

## Abstract

We investigated the effects of the administration of docosahexaenoic acid (DHA) post-traumatic brain injury (TBI) on reducing neuroinflammation. TBI was induced by cortical contusion injury in Sprague Dawley rats. Either DHA (16 mg/kg in dimethyl sulfoxide) or vehicle dimethyl sulfoxide (1 ml/kg) was administered intraperitonially at 5 min after TBI, followed by a daily dose for 3 to 21 days. TBI triggered activation of microglia or macrophages, detected by an increase of Iba1 positively stained microglia or macrophages in peri-lesion cortical tissues at 3, 7, and 21 days post-TBI. The inflammatory response was further characterized by expression of the proinflammatory marker CD16/32 and the anti-inflammatory marker CD206 in Iba1^+^ microglia or macrophages. DHA-treated brains showed significantly fewer CD16/32^+^ microglia or macrophages, but an increased CD206^+^ phagocytic microglial or macrophage population. Additionally, DHA treatment revealed a shift in microglial or macrophage morphology from the activated, amoeboid-like state into the more permissive, surveillant state. Furthermore, activated Iba1^+^ microglial or macrophages were associated with neurons expressing the endoplasmic reticulum (ER) stress marker CHOP at 3 days post-TBI, and the administration of DHA post-TBI concurrently reduced ER stress and the associated activation of Iba1^+^ microglial or macrophages. There was a decrease in nuclear translocation of activated nuclear factor kappa-light-chain-enhancer of activated B cells protein at 3 days in DHA-treated tissue and reduced neuronal degeneration in DHA-treated brains at 3, 7, and 21 days after TBI. In summary, our study demonstrated that TBI mediated inflammatory responses are associated with increased neuronal ER stress and subsequent activation of microglia or macrophages. DHA administration reduced neuronal ER stress and subsequent association with microglial or macrophage polarization after TBI, demonstrating its therapeutic potential to ameliorate TBI-induced cellular pathology.

## Introduction

Traumatic brain injury (TBI) is a serious medical condition resulting from damage to the brain due to an external mechanical force (Xiong et al., 2013). TBI is the leading cause of death and disability for people under the age of 45 years, and it is estimated that nearly 10 million deaths or hospitalizations occur annually as a result of TBI (Langlois et al., 2006). As an intricate disease process, TBI is characterized by both primary and secondary injury phases (Masel and DeWitt, 2010). The primary injury associated with TBI occurs immediately after the mechanical disruption of brain tissue, whereas secondary injury subsequently develops over an elongated time course and triggers a complex cascade of cellular events that ultimately leads to progressive neuronal cell death (Hernandez-Ontiveros et al., 2013). Accordingly, TBI is a risk factor for the pathogenesis of chronic neurodegenerative disorders, such as Alzheimer’s disease and Parkinson’s disease (Hernandez-Ontiveros et al., 2013; Jordan, 2013).

Neuroinflammation plays a key role as a secondary injury mechanism after TBI, and it has been attributed to neurodegeneration and neurological impairments (Kumar and Loane, 2012). The neuroinflammatory response following trauma is both detrimental and beneficial, contributing to secondary brain damage but also facilitating neurorepair (Woodcock and Morganti-Kossmann, 2013). Microglia, as the resident tissue macrophages of the central nervous system, are responsible for the manifestation of inflammation post-TBI (Turtzo et al., 2014). Microglia or macrophages are vital in the removal of cellular debris from sites of injury and are modulators of inflammation, cell survival, and cell death (Liu et al., 2010). As highly plastic cells, microglia or macrophages can assume diverse phenotypes in response to a wide variety of microenvironmental signals to polarize toward a specific functionality. Microglia or macrophages, however, possess a double-edged sword function in that they can mount both pro-survival and pro-death actions after a sustained TBI (Hernandez-Ontiveros et al., 2013). As such, TBI is considered a chronic and multifaceted inflammatory disease (Smith et al., 2013). Modulating the phenotypic conversion of microglia or macrophages to preserve the neuroprotective attributes, while minimizing the neurotoxic functions, is key in the development of TBI therapeutics.

Docosahexaenoic acid (DHA; 22:6), the most abundant omega-3 fatty acid within the central nervous system, is a major structural component of phospholipids in the plasma membranes of neurons (Stillwell and Wassall, 2003; Stillwell et al., 2005). In a variety of neuropathological conditions and neurodegenerative diseases, the presence of oxidative stress and proinflammatory responses is well documented (Adibhatla and Hatcher, 2007; Uttara et al., 2009). Studies have demonstrated that DHA has neuroprotective roles against oxidative stress through the intracellular scavenging of free radicals (Morgane et al., 1993; Fedorova et al., 2007; Kaur et al., 2008; Shimazawa et al., 2009). DHA is metabolized into bioactive derivatives, namely resolvins and protectins, which can resolve inflammation and facilitate neuroprotection (Mukherjee et al., 2004). In a juvenile rat cortical contusion injury (CCI) model, oral fish oil treatment was shown to attenuate disruption of the blood–brain barrier and gene expression of *Mmp9*, a key mediator in immune cell recruitment (Russell et al., 2014); however, the mechanism through which DHA regulates microglial or macrophage function and exerts neuroprotection after TBI remains incompletely studied.

In a rat CCI model, we recently documented that post-TBI administration of DHA reduces ER stress, abnormal protein accumulation, and improves recovery of sensorimotor function (Begum et al., 2014). In the current study, we further investigated whether ER stress-associated inflammation plays a role in neuroinflammation and microglial or macrophage activation. We report here that the post-TBI administration of DHA decreases neuroinflammation in part by reducing neuronal ER stress and ER stress-associated inflammation, while also stimulating a reparative microglial or macrophage phenotype. Our findings suggest that DHA could serve as an easily accessible and inexpensive supplement that can combat neuroinflammation and promote brain tissue repair after TBI.

## Materials and Methods

### Materials

Cis-4, 7, 10, 13, 16, 19-docosahexaenoic acid (DHA) and dimethyl sulfoxide (DMSO) were from SigmaChemicals (St. Louis, MO). Rat anti-mouse CD16/32 monoclonal antibody was from BD Biosciences (Cat#553141, San Jose, CA). Goat anti-mouse CD206 polyclonal antibody was from R&D Systems (Cat#AF2535, Minneapolis, MN). Rabbit anti-mouse Iba1 polyclonal antibody was from Wako (Cat#019-19741, Richmond, VA). Mouse anti-CHOP monoclonal antibody and rabbit anti-β-actin were from Cell Signaling Technology (Cat#2895, Cat#4970S, Danvers, MA). Rat anti-LAMP-1 monoclonal antibody was from Abcam (Cat#ab25245, Cambridge, MA). Donkey anti-goat Alexa Fluor® 488-conjugated IgG, goat anti-rat Alexa Fluor® 488-conjugated IgG, donkey anti-rabbit Alexa Fluor® 546-cojugated IgG, goat anti-rabbit Alexa Fluor® 546-cojugated IgG, and TO-PRO®-3 iodide were from Invitrogen (Carlsbad, CA). Rabbit anti-nuclear factor kappa-light-chain-enhancer of activated B cells (NF-κB) p65 was from Santa Cruz Biotechnology (Cat#sc-372, Dallas, TX). Rabbit anti-TATA binding protein was from Abcam (Cat#ab63766, Cambridge, UK). Goat anti-rabbit IgG (H + L)-HRP conjugated was from Bio-Rad (Cat#172-1019, Hercules, CA). Fluoro-Jade C (FJC) was from Histo-Chem Inc. (Jefferson, AK). ELISA kits (DuoSet ELISA) for cytokine measurements were purchased from R&D Systems (Minneapolis, MN, USA).

### Animals

All studies were in compliance with the guidelines outlined in the Guide for the Care and Use of Laboratory Animals from the U.S. Department of Health and Human Services and were approved by the University of Pittsburgh Medical Center Institutional Animal Care and Use Committee.

A total of 72 male Sprague–Dawley rats (250–275 g body weight) were used in the study; 40 rats were perfused for staining analyses, 21 rats were used for immunoblotting analyses, and 11 rats were used for ELISA. Rats were group-housed (two per cage) in standard steel or wire mesh cages at room temperature (22 ± 2℃) under standard 12-hr light or dark cycles (light on at 07:00 a.m.) with free access to food and water. After injury, rats were housed in separate cages from the uninjured animals under the same conditions as described earlier.

### Surgical Procedures and TBI Induction

Rats were anesthetized initially with 4% isoflurane with a 2:1 N_2_O/O_2_ mixture in a vented anesthesia chamber. Following endotracheal intubation, rats were ventilated mechanically with a 1% to 1.5% isoflurane mixture. Animals were mounted in a stereotaxic frame on the injury device in a supine position as described before (Dixon et al., 1991), and the core body temperature was monitored continuously by a rectal thermistor probe and maintained at 37 ± 0.5℃ with a heating pad. Following a midline incision and dissection of the soft tissues, a 7-mm craniotomy was made between lambda and bregma and centered 5 mm lateral of the central suture. To control for nonspecific effects due to animal handling, anesthesia or surgery procedures, sham control animals underwent identical surgical procedures but did not receive a TBI. To induce TBI, the controlled CCI device consisting of a small (1.98 cm) bore and an impactor tip (6 mm diameter) was set to produce a tissue deformation of 3.2 mm as described previously (Dixon et al., 1991). The animals received a cortical impact through the right craniotomy at a velocity of 4 m/s. Following the surgical procedures, the anesthesia was stopped, and the animals were ventilated on 100% oxygen until spontaneous return of respiration. Animal recovery was monitored by recording the times until the return of following reflexes: corneal, pinna, tail pinch, toe pinch, and righting. After this, the rats were monitored until fully recovered from anesthesia and were returned to the housing facility.

### DHA Administration

**Figure 1.**
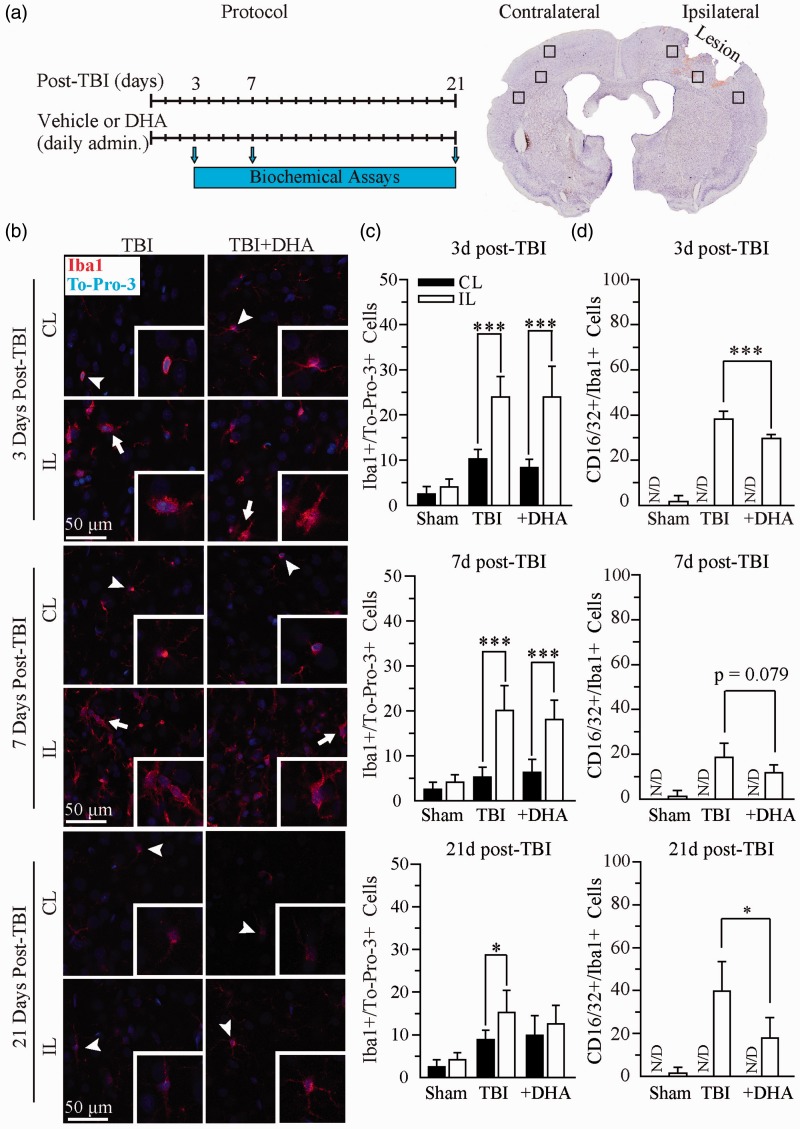
Iba1^+^ microglial or macrophage population was not reduced in the DHA-treated TBI brains. (a) Experimental protocol and location of data collection. (b) Confocal images of Iba1^+^ microglial or macrophage in the IL and CL frontal cortices at 3, 7, and 21 days post-TBI. (c) Summary data of Iba1^+^ cells/TO-PRO-3^+^ cells. Values are mean ± *SE* (*n* = 4). (d) Summary data of CD16/32^+^/Iba1^+^ immunopositive cells. Values are mean ± *SE* (*n* = 4). *p < .05, **p < .01, ***p < .001, N/D = no data, one-way ANOVA tests used.

TBI or TBI + DHA animals received an initial DMSO (1 ml/kg, i.p.) or DHA injection (16 mg/kg in DMSO, i.p.) at 5 to 15 min after the onset of TBI and subsequent daily dose for 3, 7, or 21 days after TBI, as illustrated in Figure 1(a). Sham control rats received DMSO (1 ml/kg, i.p.) at 5 min after completion of the surgery procedure and subsequent daily dose for 3, 7, or 21 days. Biochemical assays were subsequently performed at 3 to 21 days postinjury in different groups of animals at each time point.

### Immunofluorescence Staining

Animals were deeply anesthetized and transcardially perfused with 100 ml 0.1 M phosphate buffered saline (PBS) with 50 U/ml heparin (pH 7.4) followed by 500 ml 4% paraformaldehyde (PFA) with 15% saturated picric acid in 0.1 M PBS (pH 7.4). After perfusion, brains were postfixed in 4% PFA for 12 hr, and subsequently cryoprotected with 30% sucrose in 0.1 M PBS for 24 to 36 hr at 4℃ as described earlier (Begum et al., 2014). The brains were frozen in Tissue-Tek O.C.T. compound for 10 min and cut into coronal sections (35 µm thickness) on a freezing microtome (Leica SM 2000R; Leica, Nussloch, Germany). Sections at bregma level −0.26 to −1.30 mm were selected and processed for immunofluorescence staining. Sections were rinsed in 0.1 M Tris-buffered saline (TBS, pH 7.5) for 15 min, and incubated with a blocking solution (0.1% Triton X-100 and 3% donkey serum in 0.1 M TBS) for 30 min at room temperature. Sections were then incubated with goat anti-CD206 antibody (1:200), rabbit anti-CD16/32 antibody (1:1,200), rabbit anti-Iba1 antibody (1:400), rat anti-LAMP-1 (1:100) or mouse anti-C/EBP homologous protein (CHOP) antibody (1:200) in the blocking solution overnight at 4℃. After rinsing with TBS for 30 min, sections were incubated with the following corresponding secondary antibodies in the blocking solution (1:200) for 1 h at room temperature: donkey anti-goat Alexa Fluor 488-conjugated IgG, donkey anti-rat Alexa Flour 488-conjugated IgG, and donkey anti-rabbit Alexa Fluor 546-conjugated IgG. After rinsing with TBS for 15 min, sections were incubated with TO-PRO-3 iodide in the blocking solution (1:1,000) for another 15 min at room temperature. Sections were then mounted with Vectashield mounting medium (Vector Laboratories, Burlingame, CA, USA). For negative controls, brain sections were stained with the secondary antibody only.

Fluorescent images were captured under 40× lens using a Leica DMIRE2 inverted confocal laser scanning microscope (Leica Software, Mannheim, Germany). Samples were excited at 488 nm (argon/krypton), 543 nm, and 633 nm. The emission fluorescence was recorded at 512 to 548 nm, 585 to 650 nm, and 650 to 750 nm, respectively. In a blinded manner, positively stained cells (CD16/32, CD206, or Iba1) were counted from three evenly distributed areas in each brain section of the contralateral and ipsilateral hemispheres using Metamorph Software (Downingtown, Philadelphia, PA, USA). The total number of cells in the particular area was determined by the TO-PRO-3 staining. Identical digital imaging acquisition parameters were used throughout the study. The number of cells which were positively stained with the anti-CD16/32, the anti-CD206, the anti-Iba1, or the anti-CHOP antibody in each area was normalized by the total number of cells in each particular area, and data were expressed as an average of the number of positive cells/total cells × 100. The associations between CHOP^+^ neurons and Iba1^+^ microglial or macrophages were determined by counting CHOP^+^ neurons, which show either direct contact with Iba1^+^ microglia or macrophages, with their extended processes, or are engulfed by Iba1^+^ microglia or macrophages (as shown in Figure 4).

### Skeleton Analysis of Microglia

In a blinded manner, a morphology analysis of immunofluorescent images of microglia was conducted to quantify changes in microglial processes as previously described (Morrison and Filosa, 2013). Confocal images of Iba1^+^ stained brain sections (35 µm sections) were captured under 40× lens using a Leica DMIRE2 inverted confocal laser scanning microscope (Leica Software, Mannheim, Germany) and images were Z-stacked with an interval of 3.5 µm (10 images total). The images were sampled from the ipsilateral frontal cortex near the site of injury. The Iba1 channel intensity was maximized to improve visualization of processes in Iba1^+^ cells in both TBI vehicle and TBI + DHA-treated brain sections. Immunofluorescent images were then despeckled to reduce background noise. The resulting image was converted into a binary image and then skeletonized using ImageJ software. The AnalyzeSkeleton plugin (http://imagejdocu.tudor.lu/) was applied to all skeletonized images to quantify the number of endpoints and the process length per frame. Data were expressed in the summed number of endpoints and the summed value of process length per frame.

### Brain Tissue Preparation and Immunoblotting

Rats were deeply anesthetized with pentobarbital (Nembutal, 100 mg/kg, i.p.) and decapitated. After removal of the brains, the frontal cortex tissue was dissected on a chilled ice plate and homogenized in ice-cold sucrose buffer (0.25 M sucrose, 1 mM EDTA, and 10 mM Tris-HCl, pH 7.4), supplemented with protease inhibitor cocktail (1 mg/ml each of aproinin, pepstatin, and leupeptin; 100 mg/ml phenylmethylsulfonyl fluoride and 2 mM sodium orthovanadate). The homogenized samples underwent subcellular fractionation to separate cytoplasmic and nuclear components. All steps were done at 4℃. The homogenized sample was cleaned by centrifugation at 500 *g* for 10 min and the resulting supernatant was recentrifuged at 600 *g* for 10 min. The supernatant was then centrifuged at 3,000 *g* for 10 min, and the pellet was resuspended in 300 µL of sucrose buffer with protease inhibitors and centrifuged at 300 *g* for 10 min. The resulting pellet, the nuclear fraction, was resuspended in sucrose buffer with protease inhibitors and stored at –80℃ until use. The cytosolic fraction came from the first centrifugation at 3,000 *g* as supernatant and was then centrifuged at 15,000 *g* for 45 min. The resulting supernatant was stored at –80℃ until use. The protein content was determined by a BCA protein assay kit using a 96-well micro-plate reader (Spectra Max 340; Molecular Devices).

Protein samples and prestained molecular weight markers (Bio-Rad) were separated by SDS PAGE (10% gel, Bio-Rad). The resolved proteins were electrophoretically transferred to a PVDF membrane (Millipore). The blots were incubated in 5% nonfat dry milk in TBS at 4℃. The following primary antibodies and dilutions were used for incubation at 4℃ overnight: rabbit anti-NF-κB p65 (1:1,000) and loading controls rabbit anti-TATA binding protein (1:1,000) or rabbit anti-β-actin (1:5,000) for nuclear and cytoplasmic fractions, respectively. After rinsing, the blots were incubated with horseradish peroxidase-conjugated secondary IgG for 1 hr at room temperature. Bound antibody was visualized using the enhanced chemiluminescence assay. To assure equal loading of protein content (30 µg/lane), expression of TATA binding protein or β-actin was quantified. The optical density of NF-κB was normalized to either TATA binding protein or β-actin. Relative changes in protein expression were estimated from the mean pixel density of each protein band using ImageJ software.

### Proinflammatory Cytokine Measurement

At 3 days after TBI, mice were anesthetized with 5% isoflurane vaporized in N_2_O and O_2_ (3:2) and decapitated. The contralateral and ipsilateral hemispheres were dissected, and frontal cortex tissues were cut into small pieces in a PBS buffer containing phosphatase and protease inhibitors as described previously (Shi et al., 2011). After homogenization, tissue homogenates were centrifuged at 15,000 *g* at 4℃ for 20 min, and the supernatant was collected for analysis of TNF-α and IL-1β using ELISA kits. The total protein concentration of each homogenate was determined using the BCA method. The kits were validated prior to use with brain homogenate samples. The respective capture antibodies were diluted to the working concentration in PBS. The 96-well plates were immediately coated overnight with 100 μl (TNF-α, 80 ng; IL-1β, 400 ng) per well of the diluted capture antibodies. After three rinses with washing buffer (0.05% Tween 20 in PBS, pH 7.2), the plates were blocked with blocking buffer (300 μl 1% BSA in PBS) for 1 hr. Then, 100 µL standards, or homogenate samples (0.6 mg protein) were added and allowed to incubate for 2 hr. After the incubation, 100 µL (TNF-α, 5 ng; IL-1β, 150 ng) of the biotinylated detection antibodies were added to each well and incubated for 2 hr; 100 µL of the working dilution (1:40) of the supplied streptavidin-HRP was added, and plates were allowed to incubate for 20 min. Following washing, substrate solution (1:1 mixture of color reagent A and color reagent B) was added and the reaction was terminated with stop solution. The optical density of each well at 450 nm was determined immediately using a micro-plate reader (Molecular Devices, Sunnyvale, CA, USA). A 7-point standard curve was generated every time using supplied standards for each set of samples assayed. TNF-α and IL-1β were calculated with the standard curve and expressed as pg/mg protein. Based on the standard curve, the lowest detectable limits for TNF-α and IL-1β were 3.9 pg/ml and 1.8 pg/ml, respectively.

### Fluoro-Jade C staining and Quantification

Brain sections (35 µm) at bregma level −0.26 to −1.30 mm were mounted on slides and dried on a slide warmer at 50℃ for 30 min, as described before (Begum et al., 2014). On the following day, sections were incubated with 0.06% KMnO_4_ for 15 min in the dark. After a brief rinse in ddH_2_O, the sections were stained with 0.001% FJ-C solution (resuspended in 0.1% acetic acid) for 25 min on a shaker. Sections were then rinsed in ddH_2_O followed by air-drying on the slide warmer at 50℃ for 10 min. Slides were then cleared in CitriSolv and mounted with DPX mounting medium. Whole brain FJ-C stained images were captured under 40× lens using a Leica DMIRE2 inverted confocal laser scanning microscope (Leica Software, Mannheim, Germany). To quantify FJ-C positive cells, three regions from each animal were counted in a blinded manner. Representative images were captured under 40× lens using a Leica DMIRE2 inverted confocal laser scanning microscope (Leica Software, Mannheim, Germany). In a blind manner, the total number of FJ-C-positive cells in the ipsilateral frontal cortex peri-lesion was determined in each slice by using Metamorph image-analysis software (Molecular Devices, Downington, PA).

### Statistics

Data are expressed as the group means ± standard error (*SE*) of the mean. Statistical significance was determined by paired *t*-test or one-way ANOVA using the Bonferroni post hoc test in case of multiple comparisons (SigmaStat, Systat Software, Point Richmond, CA, USA). Pearson correlation coefficient was calculated using online statistics software (Office for Research Development and Education, version 1.1.23-r7). A significance level of *p* < .05 was used for all tests.

## Results

### TBI Triggers Chronic Activation of Microglia or Macrophages

We first wanted to characterize the microglial or macrophage activation state with and without DHA treatment at several time-points post-TBI. After TBI induction, animals were randomly assigned to either vehicle or DHA treated groups (Figure 1(a)). TBI or TBI + DHA animals received an initial DMSO (1 ml/kg, i.p.) or DHA injection (16 mg/kg in DMSO, i.p.) at 5 min after the onset of TBI and a subsequent daily dose. At 3, 7, or 21 days after TBI, animals were sacrificed for the study. Data were collected from the contralateral (CL) frontal cortex and the ipsilateral (IL) frontal cortex surrounding the site of lesion. Activated microglia or macrophages were detected by expression of the marker protein ionized calcium-binding adaptor molecule 1 (Iba1). In the CL frontal cortex, Iba1^+^ cells exhibited characteristics of a surveillant or “ramified” form with a small cell body and long branched processes (arrowhead, enlarged in inset, Figure 1(b)). In the IL frontal cortex, there was a significant increase in the number of Iba1^+^ cells compared with the CL frontal cortex. The activated Iba1^+^ cells had an “amoeboid-like” appearance at 3 and 7 days after TBI (arrow, enlarged in inset). The microglial or macrophage cell underwent a morphological change in which the cell body became hypertrophic and the processes retracted, giving rise to the amoeboid-like microglial cell. At 21 days post-TBI in the IL frontal cortex, the morphology of Iba1^+^ cells remains at the transformed activated state from the surveillant control state (arrowhead, enlarged in inset). Overall, the stimulation of activated microglia or macrophages was prolonged for 21 days post-TBI.

Unexpectedly, DHA treatment had no effect on reducing the number of activated Iba1^+^ cells in the frontal cortex post-TBI (Figure 1(c)). At 3 days post-TBI, vehicle control brains and the DHA-treated brains exhibited the identical level of Iba1^+^ cells (23.9 ± 4.6% and 23.9 ± 6.8% of total cells, respectively). The increase of Iba1^+^ cells in the IL frontal cortex of vehicle control TBI brains extended to 7 days (20.1 ± 5.5%) and 21 days (15.2 ± 5.2 %) after TBI. The number of the Iba1^+^ cells also remained elevated in the IL frontal cortex in the DHA-treated TBI brains at 7 days (18.1 ± 4.3%) and 21 days (12.5 ± 4.4%).

We further analyzed microglial or macrophage activation by examining the proinflammatory M1 marker CD16/32 in Iba1^+^ cells in TBI brains. TBI triggered an elevation of the CD16/32^+^/Iba1^+^ population in the IL frontal cortex tissues at 3 days post-TBI (39.6 ± 2.6% of cells were CD16/32^+^/Iba1^+^), whereas TBI + DHA brains showed moderately reduced numbers of CD16/32^+^/Iba1^+^ (29.3 ± 1.5%, *p* < .001, Figure 1(d)). At 7 days post-TBI, the CD16/32^+^ and Iba1^+^ microglial or macrophage cells were lower but remained elevated (19.9 ± 5.9% of cells) in the IL frontal cortex. In contrast, CD16/32^+^/Iba1^+^ cell numbers in the DHA-treated brains were further decreased but did not reach significance (11.2 ± 4.0%, *p* = .079). The pattern of CD16/32^+^/Iba1^+^ cells in the vehicle control TBI brains and DHA-treated brains remained significantly elevated at 21 days (39.8 ± 13.9% vs. 17.9 ± 9.6%, *p* < .05, Figure 1(d)). Supplemental Figure 1 illustrates that CD16/32^+^/Iba1^+^ microglial or macrophage morphology at 3, 7, and 21 days post-TBI was characteristic of the “amoeboid-like” appearance. Collectively, DHA treatment attenuated the proinflammatory CD16/32^+^/Iba1^+^ cell population post-TBI.

### DHA-Treated Brains Exhibited Faster Recovery of Surveillant State Morphology Post-TBI

**Figure 2.**
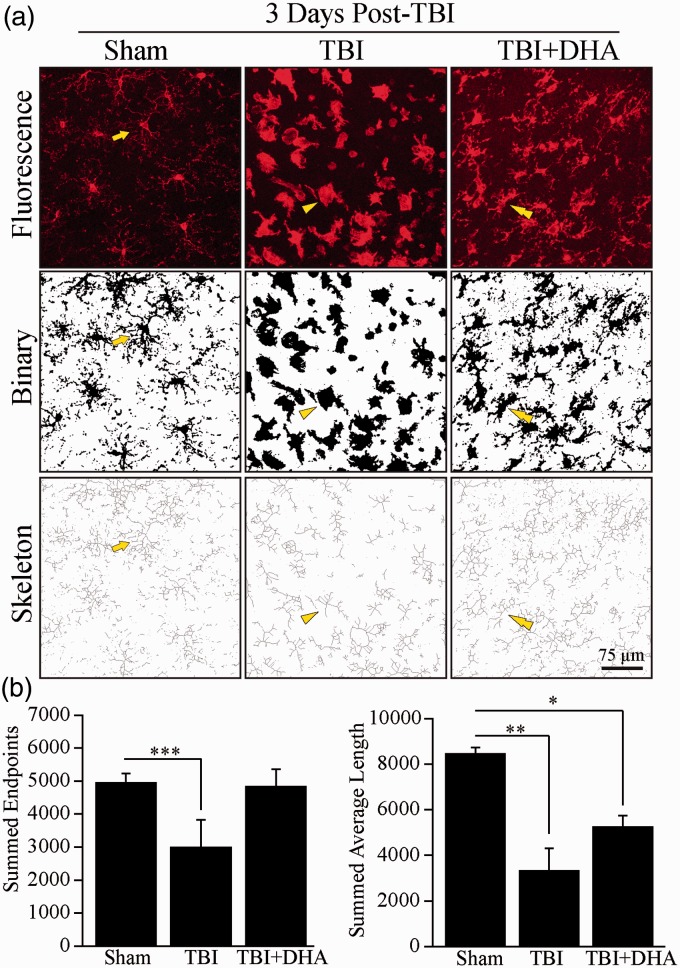
DHA treatment increased microglial ramification post-TBI. (a) Confocal Z-scan intensified images show Iba1^+^ microglia or macrophages in the IL frontal cortices at 3 days post-TBI. Binary images clarify the boundaries of microglial or macrophage cell morphology. Skeletonized images show the endpoints and length of each microglial or macrophage cell as individual pixels. Arrow: Ramified or surveillant microglia/macrophage. Arrowhead: Activated microglia or macrophage. Double Arrowhead: Microglia or macrophage reverting from hypertrophic and retracted processes morphology into the ramified or surveillant state. (b) Summary data of summed endpoints and summed average length of skeletonized images of Iba1^+^ microglia or macrophages. Values are mean ± *SE* (*n* = 4). *p < .05, **p < .01, ***p < .001, one-way ANOVA test used.

We then quantified changes of microglial or macrophage morphology in cortical brain tissue 3 days post-TBI. Representative confocal Z-scan images were taken and intensified to fill in the intricacies of microglial or macrophage processes (Figure 2(a)). The confocal images were converted into a binary form and then skeletonized for analysis of cellular morphology of Iba1^+^ cells. In the sham tissue, the ramified microglial or macrophage morphology was characteristic of a small cell body and long branching cellular extensions with high values of the summed number of endpoints (4,946.7 ± 287.4) and summed average length of processes (8,452.3 ± 273.2, Figure 2(b)). At 3 days post-TBI, Iba1^+^ microglial or macrophage morphology became hypertrophic, resulting in a 39.5% (*p* < .001) reduction in the number of endpoints and a 60.8% (*p* < .01) decrease in the summed average length of microglial or macrophage processes (Figure 2(b)). However, DHA treatment somewhat restored the ramification of Iba1^+^ cells (Figure 2(a)). DHA-treatment induced a morphological shift away from the amoeboid-like shape and toward the classic ramified morphology with cellular extensions protruding from the cell body (Figure 2(a), double arrowhead). These morphological observations suggest that microglia or macrophages began to transform back into a surveillant or restorative phenotype. Ultimately, DHA demonstrated its effects in changing the morphology of microglia or macrophages back into a ramified or surveillant state after TBI, suggesting a potential shift in phenotype.

### DHA Treatment Promotes a Resolving Phenotype Polarization of Microglia or Macrophages After TBI

**Figure 3.**
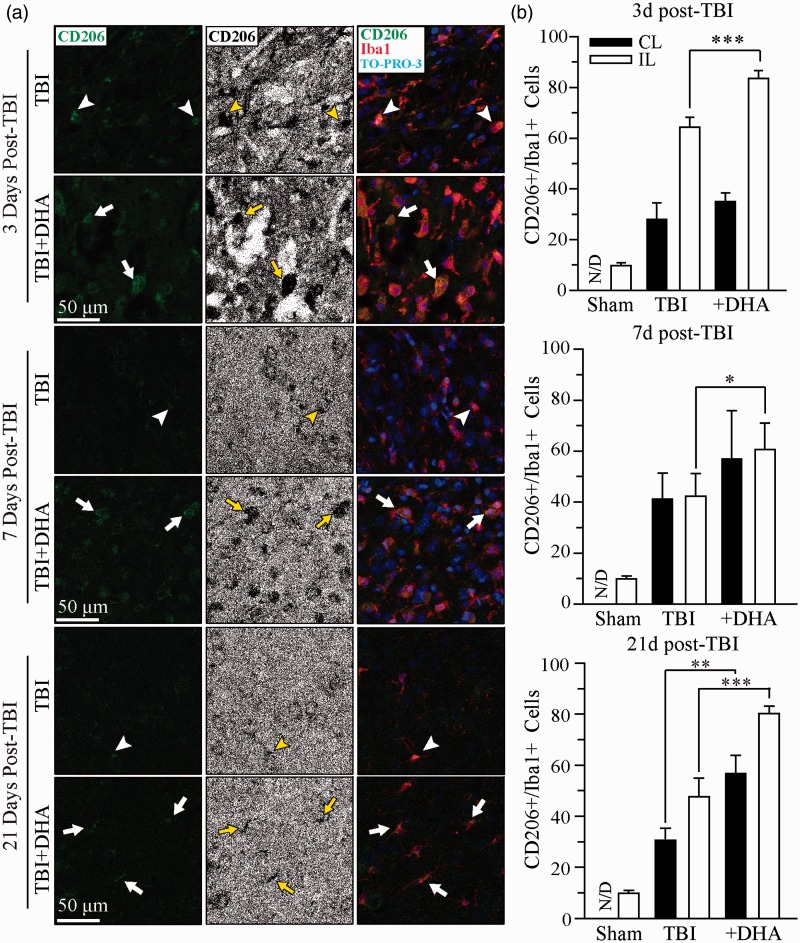
DHA treatment increases CD206^+^ microglial or macrophage population in post-TBI brains. (a) Confocal images show Iba1^+^ and CD206^+^ microglia or macrophages in the IL frontal cortices at 3, 7, and 21 days post-TBI. Confocal images of CD206^+^ microglia or macrophages were binaried for clarity. Arrowhead: colocalization of Iba1^+^ and CD206^+^ immunostaining (red and green). Arrow: increased CD206^+^ expression in microglia or macrophages in the DHA-treated TBI tissues. (b) Summary data of CD206^+^/Iba1^+^ immunopositive cells. Values are mean ± SE (*n* = 4). *p < .05, **p < .01, ***p < .001, N/D = no data, one-way ANOVA test used.

We further investigated whether DHA promotes the resolving phenotype of microglial or macrophage activation in Iba1^+^ cells after TBI by examining expression of CD206, an anti-inflammatory M2 phagocytic microglial or macrophage marker. TBI triggered an elevation of CD206^+^/Iba1^+^ cells in the IL frontal cortex at 3 days post-TBI (Figure 3, arrowhead). Moreover, the morphology of the CD206^+^/Iba1^+^ cells at 3 and 7 days was characteris or macrophages. The percentage of CD206^+^/Iba1^+^ cells in the IL frontal cortex of non-treated brains was 62.4 ± 5.4% at 3 days post-TBI. CD206^+^ expression in the Iba1^+^ cells slightly decreased at 7 and 21 days post-TBI to 45.3 ± 6.9% and 47.6 ± 7.3%, respectively. In contrast, the administration of DHA post-TBI significantly increased expression of the CD206^+^/Iba1^+^ cells throughout 3 to 21 days post-lesion (Figure 3(b); 3 days, 83.3 ± 2.7%, *p* < .001; 7days, 65.1 ± 2.2%, *p* < .05; 21days, 80.2 ± 2.9%, *p* < .001). At 21 days, however, the morphology transformed to the surveillant microglial or macrophage state. These data further suggest that DHA may facilitate microglial or macrophage expression of biochemical markers associated with reparative actions.

**Figure 4.**
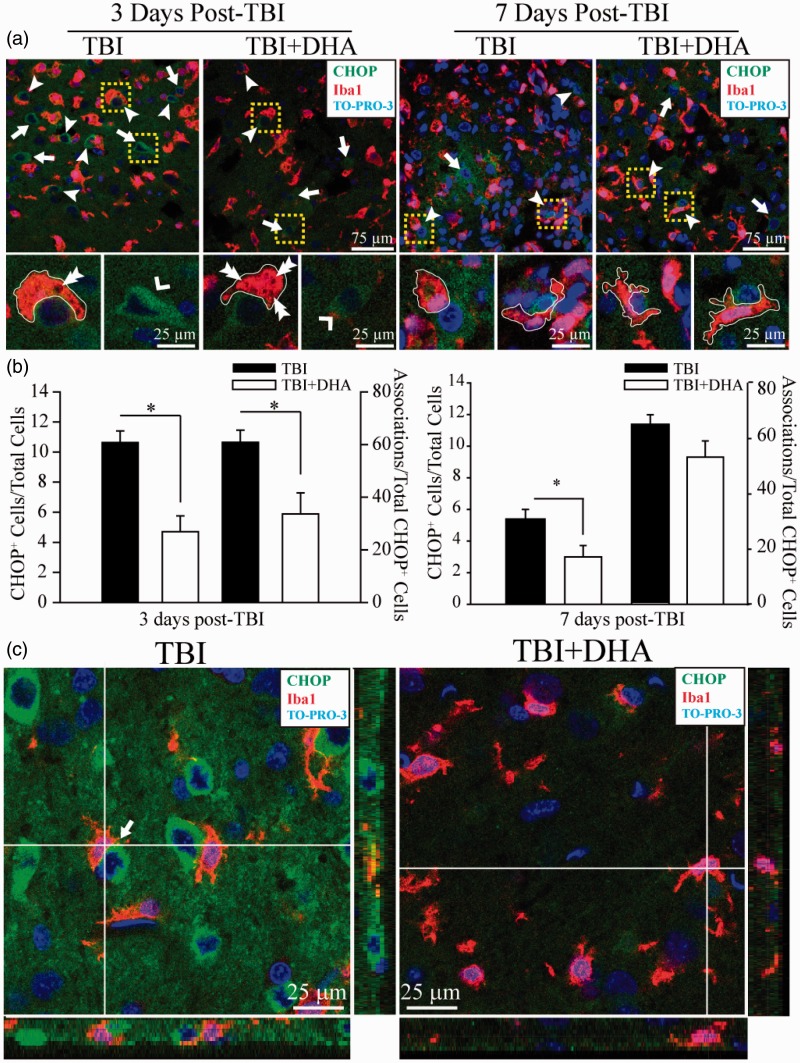
DHA reduces TBI-mediated ER stress and microglial or macrophage activation. (a) Confocal images show expression of ER stress marker protein CHOP and microglial or macrophage marker Iba1 in the IL frontal cortex at 3 and 7 days post-TBI. Arrow: Increased expression of CHOP in IL frontal cortex tissue. Arrowhead: Association of CHOP^+^ neurons with Iba1^+^ microglia or macrophages. Double Arrowhead: Vacuoles in phagocytizing microglia or macrophages. Yellow Box: Iba1^+^ microglia or macrophage in close contact with CHOP^+^ neurons. Outlines of microglial or macrophage morphology demonstrate a reramification with DHA treatment at 7 days post-TBI. (b) DHA treatment significantly reduced the expression of CHOP in neurons (*n* = 5) and reduced the number of CHOP^+^/Iba1^+^associations (*n* = 5) at 3 days post-TBI. Values are mean ± *SE* *p < .05, paired *t*-test used. (c) Z-sectioning images demonstrating interactions between CHOP^+^ neurons (green) with Iba1^+^ microglia or macrophages (red). Arrow: colocalization.

### TBI Induces an Association Between Neuronal ER Stress and Microglial or Macrophage Activation

ER stress-associated inflammation plays an important role in triggering several inflammation pathways (Hotamisligil, 2010). We further investigated whether there is an association between neuronal ER stress and microglial or macrophage activation. TBI triggered an increase in the expression of ER stress marker protein CHOP in neurons (Figure 4(a), arrowhead). Additionally, CHOP^+^ neurons were surrounded by Iba1^+^ microglia or macrophages in the IL frontal cortex at 3 days post-TBI (Figure 4(a), dashed box). With the administration of DHA post-injury, there was a significant reduction in the number of CHOP^+^ neurons in the IL frontal cortex as well as a decreased association between CHOP^+^ neurons and Iba1^+^ microglial or macrophages (Figure 4(b), *p* < .05). Microglial or macrophage morphology was characteristically enlarged and hypertrophic at 3 days post-TBI. In contrast, the DHA-treated tissue showed an increase in ramification and a thinning of the cell body. It was qualitatively observed that DHA treatment caused an increase in the number of vacuoles associated with phagocytic activity in microglia or macrophages (Figure 4(a), double arrowhead). These phenomena are further illustrated in Z-sectioning images in Figure 4(c) that demonstrate CHOP^+^ neurons and Iba1^+^ microglial or macrophage cells are clearly associated by physical contacts (colocalization of green and red signals) at 3 days post-TBI. However, such relationships are not readily observable in TBI + DHA brains. Taken together, these data suggest that DHA reduces ER stress in neurons, which may in turn serve to reduce microglial or macrophage activation post-TBI.

We also examined phagocytosis state by evaluating expression of a lysosomal marker LAMP-1. Z-sectioning confocal images in Supplemental Figure 2(a) show abundant expression of LAMP-1^+^ neurons (arrow), which were surrounded by Iba1^+^ cells in the IL frontal cortices of the TBI brains at 3 days post-TBI. In contrast, DHA treatment reduced the expression of lysosomal marker LAMP-1 post-TBI (Supplemental Figure 2(a) and (b)). These data suggest DHA-mediated, reparative microglial or macrophage activation may in part derive from reducing neuronal ER stress and lysosomal activation.

**Figure 5.**
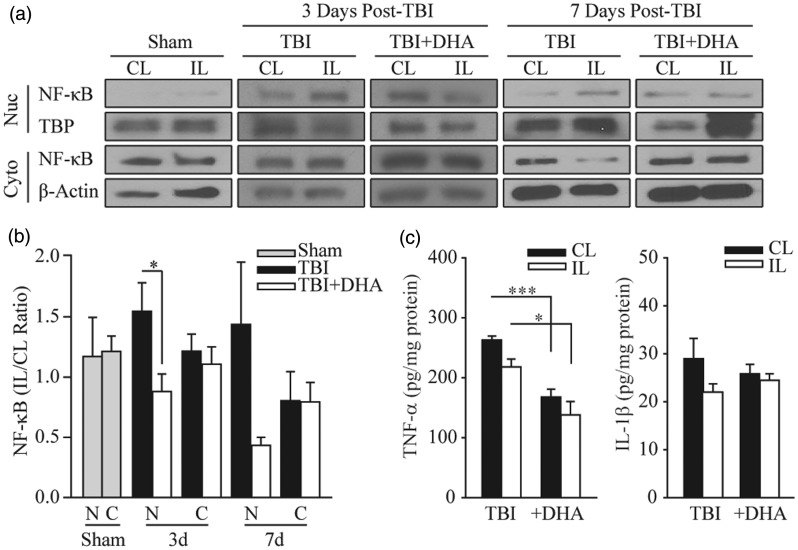
DHA-treated rats exhibit reduced NF-κB translocation to the nucleus after TBI. (a) Representative immunoblots of NF-κB nuclear and cytosolic expression in the CL and IL frontal cortices of TBI and TBI + DHA animals at 3 days and 7 days post-TBI. The same blot was probed with either antibody against nuclear TATA binding protein (TBP) or against β-actin as a loading control. (b) Summary data expressed as NF-κB expression in nuclear and cytosolic fractions (IL/CL). Values are mean ± SE (sham, *n* = 3; 3 days post-TBI or TBI + DHA, *n* = 6; 7 days post-TBI or TBI + DHA, *n* = 3). Paired *t*-test used. (c) Analysis of TNF-α and IL-1β in the CL and IL cortical tissues with ELISA. Values are mean ± *SE* (*n* = 5–6). *p < .05, ***p < .001, paired *t*-test used.

To further investigate ER stress-associated inflammation and microglial or macrophage activation, we probed expression of the proinflammatory transcription factor NF-κB in cytosolic and nuclear cell fractions of TBI brains. Western blot analysis revealed that the majority of NF-κB protein was located in the cytosolic subcellular fraction and only a small portion was in the nuclei (Figure 5(a)). TBI triggered an increased expression of NF-κB in the IL cortical tissues when compared with the CL samples. The DHA-treated rats exhibited a significant reduction in NF-κB nuclear translocation at 3 days post-TBI (Figure 5(b), *p* < .05). Nuclear levels of NF-κB remain elevated at 7 days post-TBI. Although DHA-treatment reduced the nuclear translocation of NF-κB, but it did not reach statistical significance (*p* = .07). Taken together, these data imply that DHA administration may reduce microglial or macrophage activation via reduction of NF-κB nuclear translocation.

We then determined the expression of proinflammatory cytokines TNF-α and IL-1β in vehicle control and DHA-treated TBI brains. As shown in Figure 5(d), there was no elevation of TNF-α and IL-1β in IL cortical tissues 3 days post-TBI. In fact, a 36.7% reduction in TNF-α content was detected in the IL cortex of TBI brains (*p* < .05). The absence of an increase in TNF-α and IL-1β expression by 3 days after lesion is consistent with a previous report that demonstrated the peak time for proinflammatory cytokine expression was at 1 to 3 hr post-TBI. This same study has shown that proinflammatory cytokine expression has rapidly declined to the basal levels by 24 to 72 hr post-injury (Vitarbo et al., 2004). Interestingly, the DHA-treated brains exhibited significantly lower amounts of TNF-α in both CL and IL cortices when compared with TBI brains (Figure 5(d), *p* < .001). This effect is likely attributed to the anti-inflammatory functions of DHA (Begum et al., 2013).

### TBI Triggers a Positive Correlation Between Neurodegeneration and Neuronal ER Stress

**Figure 6.**
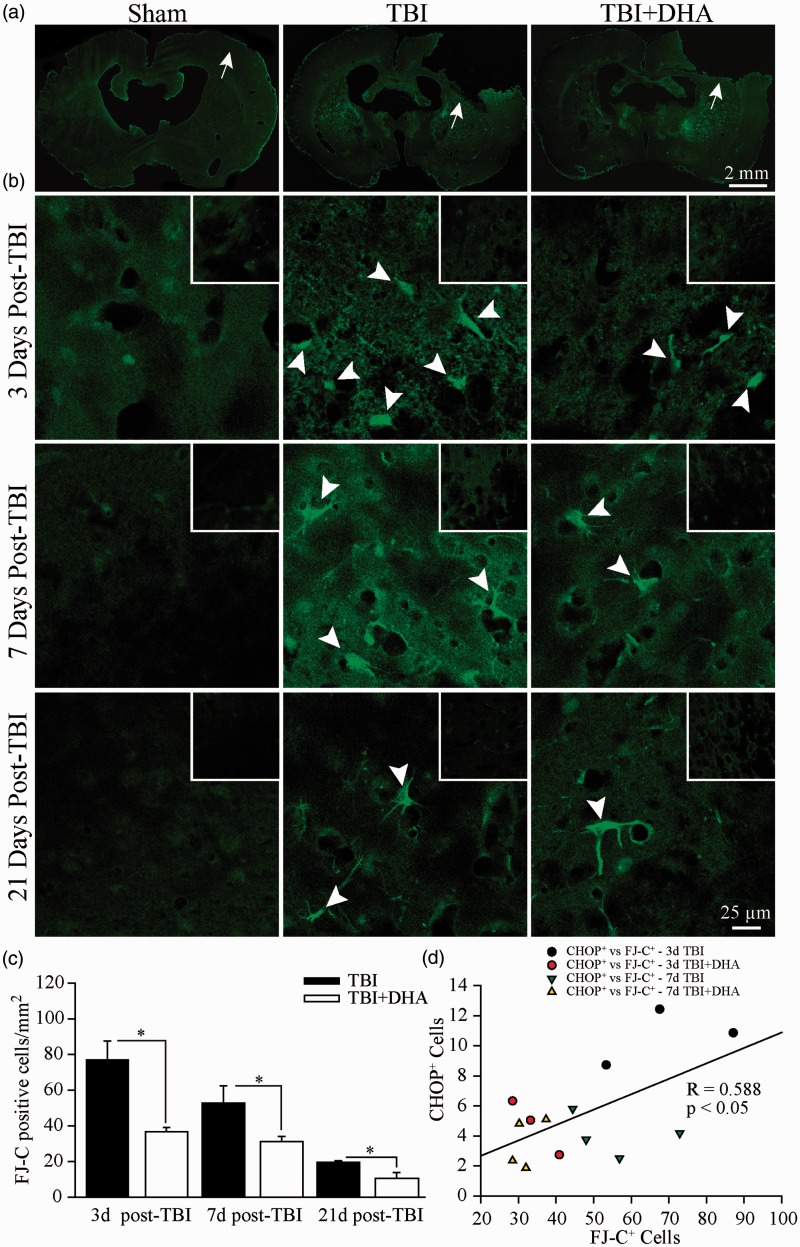
Positive correlation between reduced neuronal damage and ER stress in the DHA-treated brains. (a) Microscopic fluorescent images of sham, TBI, and TBI + DHA brains stained with FJ-C. Arrow: site of surgery. (b) Representative confocal images show FJ-C stained neurons in the IL frontal cortex peri-lesion at 3 to 21 days post-TBI. Inset: CL frontal cortex. Arrowhead: FJ-C positive degenerating neurons. Inset shows CL frontal cortex tissue at the same location. (c) Summary data of FJ-C positive cells per mm^2^. Values are mean ± *SE* (*n* = 4). (d) Correlation analysis of CHOP^+^ cells versus FJ-C^+^ cells (*R* = .588, *p* < .05, *n* = 14). *p < .05, one-way ANOVA test used.

To determine the effect of TBI and DHA on neuronal survival, we performed FJ-C staining to quantify neuronal degeneration in the IL frontal cortex at 3 to 21 days post-TBI (Figure 6(a)). Higher magnification revealed an increase in the FJ-C positive neurons confined to the peri-lesion area (Figure 6(b), arrowhead) of TBI brains at 3 to 21 days post-TBI. In contrast, no such neurodegeneration was observed either in sham brain or the CL cortex of TBI brains at 3, 7, and 21 days (Figure 6(b), inset). Summary data are shown in Figure 6(c). At 3 days post-TBI, the number of FJ-C positive cells/mm^2^ was reduced from 77.3 ± 10.6 in TBI tissue to 36.3 ± 3.3 in DHA-treated TBI tissue (*p* < .05). Moreover, at 7 days post-TBI, there was a reduction from 55.6 ± 6.3 to 32.0 ± 1.9 FJ-C positive cells/mm^2^ in TBI + DHA brain tissue (*p* < .01). Lastly, at 21 days post-TBI the DHA-treated brain tissue had 11.9 ± 2.1 FJ-C positive cells/mm^2^ in comparison with 19.6 ± 1.0 FJ-C positive cells/mm^2^ in TBI tissue (*p* < .05). DHA treatment significantly reduced the FJ-C positive degeneration at 3, 7, and 21 days post-TBI, indicating that DHA administration post-TBI enhances neuroprotection.

We further analyzed a correlation between neurons undergoing ER stress (CHOP^+^) and degenerating neurons (FJ-C^+^). The results showed a positive correlation of *R* = 0.588 and *p* < .05, suggesting that ER stress plays a role in neurodegeneration post-TBI (Figure 6(d)). Therefore, ER stress may contribute to neuronal death via stimulating microglial or macrophage activation and their consequent proinflammatory actions.

## Discussion

### DHA and the Activation of Microglia or Macrophages in TBI

The activation of microglia or macrophages and their transformation between different phenotypes is not well understood in TBI brains. Studies have shown that microglia or macrophages can contribute to injury via proinflammatory responses after lesion, yet at the same time, microglia or macrophages are vital for facilitating neuroreparative processes (Taylor and Sansing, 2013). In our study, we detected elevated Iba1^+^ cell numbers with increased proinflammatory CD16/32^+^ M1 phenotype at 3 days post-TBI. The sustained expression of the proinflammatory marker CD16/32 in TBI brains remained for 21 days post-TBI. Simultaneously, there was a heightened stimulation of CD206 expression after TBI, a marker for the reparative phenotype, suggesting that reparative processes are triggered after the insult of trauma. DHA treatment attenuated the expression of the M1-associated microglial or macrophage marker CD16/32 at 3 to 21 days post-TBI in the IL frontal cortex and significantly increased the expression of the anti-inflammatory M2 marker CD206 at 3 to 21 days after TBI. Our data are consistent with previous reports that have shown a shift of M2-to-M1 phenotype over time (David and Kroner, 2011; Hu et al., 2012; Wang et al., 2013). We detected a reduction in the levels of the M2 phenotype and sustained M1 phenotype in the vehicle control group. Overall, our data align with the notion that chronic activation of reactive microglia is destructive over time. Additionally, we observed that microglial or macrophage morphology shifted away from the surveillant, ramified state to an amoeboid-like shape at 3 days post-TBI. DHA treatment retained microglial or macrophage morphology characteristic of the surveillant state that suggests a potential transformation in functional orientation. DHA-treated brains exhibited some microglia or macrophages with a hypertrophic cell body and increased phagocytic vacuoles, indicative of anti-inflammatory actions such as the phagocytosis of cellular debris and injured neurons undergoing ER stress.

These findings suggest that DHA may be able to facilitate the polarization of microglia or macrophages into a phenotype associated with anti-inflammatory and reparative actions; however, morphology is not always indicative of functional orientation. As such, additional studies are needed to study gene profiles of pro- and anti-inflammatory cytokines in TBI and DHA-treated brains to further investigate the correlation between morphological changes of microglia or macrophages and neuroinflammation.

Despite the beneficial effects of DHA administered at 5 to 15 min post-TBI, this treatment regimen is not suitable for translation into clinical therapy development. Therefore, another study is needed to determine whether delayed and sustained post-TBI administration of DHA has similar effects on reducing ER stress and altering inflammation processes.

### ER Stress-Associated Inflammation Pathways and Microglial or Macrophage Activation

We also observed an association between neuronal ER stress and microglial or macrophage activation in this study. There was an increase in the expression of the neuronal ER stress marker CHOP in the IL frontal cortex post-TBI. Neurons expressing CHOP had a tendency to be surrounded by activated Iba1^+^ microglial or macrophage cells, suggesting that microglial or macrophage activation may in part respond to neuronal ER stress. It has been suggested that microglia or macrophages are inappropriately initiating phagocytosis of stressed neurons, such as CHOP^+^ cells, rather than facilitating cell suicide (Lenz and McCarthy, 2014). DHA treatment reduced the occurrence of these associations between neurons undergoing ER stress and activated microglia or macrophages, implying that DHA may play a role in facilitating microglia or macrophages into a more permissive phenotype. Accordingly, we found that there was an increase in the ramification of microglia or macrophages phagocytizing CHOP^+^ neurons in the DHA-treated brains, supporting the notion of DHA-mediated anti-inflammatory actions.

Under conditions conducive to ER stress, the activated prongs of the unfolded protein response share intersections with other inflammatory and cell stress signaling systems, namely the NF-κB pathway that is both necessary for acute adaptive cell survival and detrimental when chronically stimulated (Hotamisligil, 2010). NF-κB in the cytoplasm exists in its inactivated form and translocation to the nucleus results in its activation and subsequent regulation of proinflammatory gene transcription. Our data show a significant reduction of nuclear NF-κB translocation and a visible retention of NF-κB in the cytoplasm in the IL cortex of DHA-treated brain tissues. This data, however, is for whole brain lysate and thus cannot rule out reduction is happening in other cell types besides microglia or macrophages. In our previous study, we demonstrated that the administration of DHA post-TBI attenuated PERK, a prong of the unfolded protein response, in a rat model of CCI (Begum et al., 2014). The shutdown of protein synthesis by PERK results in the release of NF-κB from its inhibitor IκB, allowing NF-κB translocation to the nucleus where proinflammatory genes encoding for cytokines like TNF-α and IL-1 are expressed (Hotamisligil, 2010). Ultimately, our data demonstrate that DHA-mediated reduction of ER stress likely attenuates PERK expression and the subsequent inhibition of NF-κB translocation to the nucleus and may play a role in reducing neuroinflammation after TBI.

The DHA-mediated reduction of ER stress prevents injured neurons from committing to cellular death pathways, which further compounds with a reduction in microglia or macrophage inflammatory responses to brain injury. In light of the effects of DHA on reducing lysosomal marker protein LAMP-1 in neurons after TBI (Supplemental Figure 2), follow-up studies may include to investigate downstream mediators in the unfolded protein response or ER stress pathways (proteasome-mediated protein degradation, autophagy, etc).

### DHA Attenuates ER Stress and Facilitates Reparative Microglial or Macrophage Phenotype Polarization

Interests in the effects of DHA on microglial or macrophage polarization in different contexts are growing. Previous studies have shown that after TBI, the metabolism of DHA into bioactive docosanoids, such as the neuroprotectins and resolvins, resulted in the inhibition of inflammatory cell activation and migration, upregulation of anti-apoptotic molecules, and expression of receptors involved in reparative processes (Hasadsri et al., 2013). Additionally, a DHA-enriched diet was shown to prevent microglial activation in a mouse stroke model, reduce ischemic lesion size, and increase levels of the anti-apoptotic molecules Bcl-2 in the CNS (Lalancette-Hebert et al., 2011). A study by Antonietta et al. (2012) found that DHA inhibited the production of proinflammatory cytokines associated with the M1 polarized microglia but did not induce an alternative anti-inflammatory microglial phenotype in cell culture. A recently published study demonstrated the protective effects of resolvins in a diffuse brain injury mouse model that displayed a higher amount of ramified microglia and a lower proportion of activated rod microglia—a unique morphology observed after neurological injury—in the cortex, suggesting a decrease in injury-induced activation (Harrison et al., 2015).

In our study, we detected reduced neuronal ER stress but not Iba1^+^ microglial or macrophage cell numbers in DHA-treated brains. The differential effects of DHA are not well understood. One possible explanation for this phenomenon is that microglial or macrophage activation is not driven by a simple “all-or-none” mechanism. The activation and polarization of microglial or macrophage cells are two fundamentally distinct, yet intertwined, events and are induced by multiple factors from injured neurons as well as astrocytes after TBI (Ziebell et al., 2012). It would not be an ideal situation during post-TBI cellular repair processes for cells to completely shut down microglial or macrophage activation. Instead, we speculate that with its pleiotropic functions DHA not only attenuates neuronal ER stress but also simultaneously activates other cellular pathways to transform and modulate activated Iba1^+^ cells by increasing expression of the anti-inflammatory phenotypes for tissue repair.

In summary, we report here that TBI triggers profound and sustained neuroinflammatory events, including the activation of microglia or macrophages, neuronal ER stress, and neuronal degeneration. The administration of DHA attenuates the destructive proinflammatory phenotype of microglia or macrophages, while simultaneously increasing reparative microglial or macrophage morphology and phenotype post-TBI. DHA-treatment reduced neuronal ER stress and associated microglia or macrophage activation. Additionally, DHA treatment decreased nuclear translocation of NF-κB after TBI. Our study is the first to suggest that administration of DHA alters microglial or macrophage morphology and activation of phenotypes post-TBI, representing a potential dietary supplement to combat neuroinflammation. DHA supplementation may be useful for the development of a therapeutic agent that can suppress proinflammatory actions, while promoting neuroprotective and anti-inflammatory effects.

## Summary Statement

The administration of docosahexaenoic acid after TBI in a rat model of cortical contusion injury reduces neuronal endoplasmic reticulum stress and its association with activated microglia or macrophages, alters microglial or macrophage polarization, and decreases neurodegeneration.

## Supplementary Material

Supplementary material
