# Supplementary figures and images for: Administration of DHA Reduces Endoplasmic Reticulum Stress-Associated Inflammation and Alters Microglial or Macrophage Activation in Traumatic Brain Injury
Source: ASN Neuro. 2015 Dec 16;7(6):1759091415618969. doi: 10.1177/1759091415618969 (PMC4710127; doi:10.1177/1759091415618969)

Supplemental Figures

A

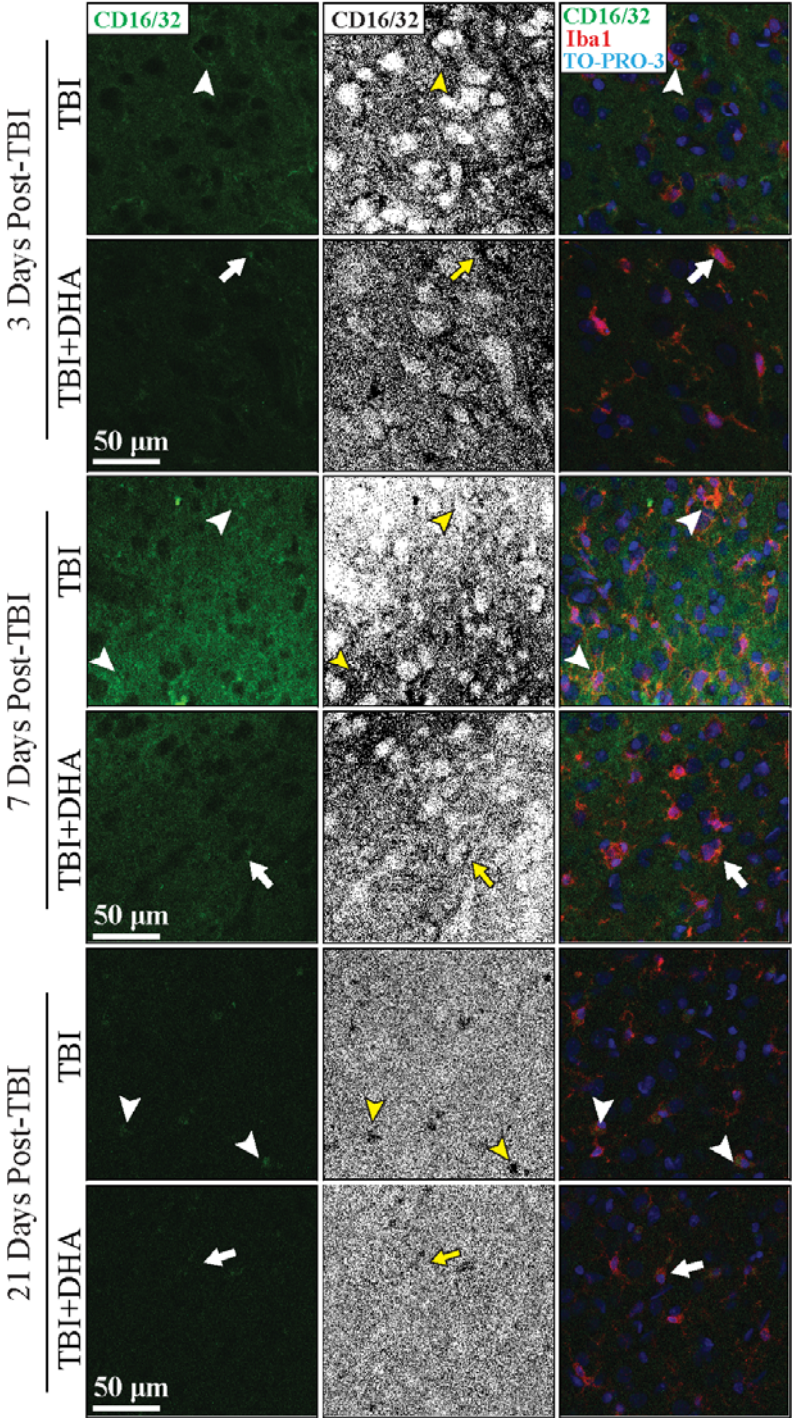

Supplemental Figure 1

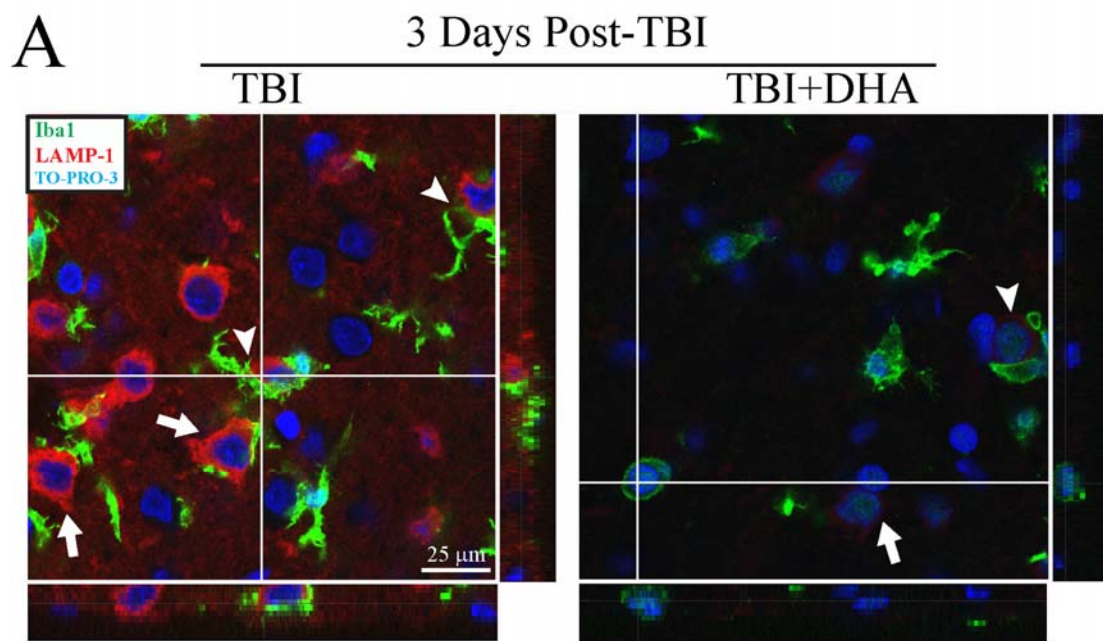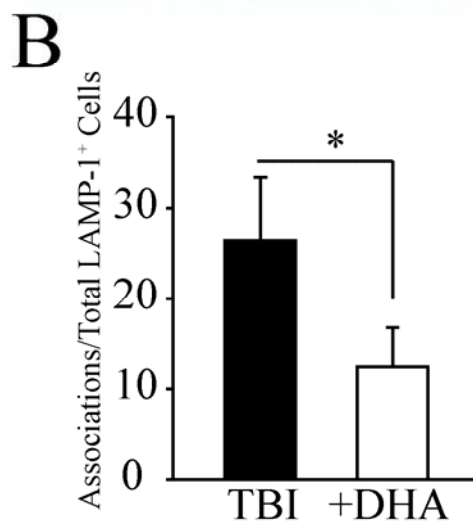

Supplemental Figure 2

Supplement: Supplementary material [file Supplemental_Figures.pdf]
